# Role of TGF-Beta Signaling in Beta Cell Proliferation and Function in Diabetes

**DOI:** 10.3390/biom12030373

**Published:** 2022-02-26

**Authors:** Hong-Lian Wang, Li Wang, Chang-Ying Zhao, Hui-Yao Lan

**Affiliations:** 1 Research Center for Integrative Medicine, The Affiliated Traditional Medicine Hospital of Southwest Medical University, Luzhou 646000, China; honglianwang@swmu.edu.cn (H.-L.W.); wangli120@swmu.edu.cn (L.W.); 2 School of Clinical Medicine, Chengdu University of Traditional Chinese Medicine, Chengdu 611137, China; 3 Department of Endocrinology, The Affiliated Traditional Medicine Hospital of Southwest Medical University, Luzhou 646000, China; zhaocy@swmu.edu.cn; 4 Department of Medicine and Therapeutics, Li Ka Shing Institute of Health Sciences, The Chinese University of Hong Kong, Hong Kong 999077, China; 5 Guangdong Academy of Sciences, Guangdong Provincial People’s Hospital Joint Research Laboratory on Immunological and Genetic Kidney Diseases, The Chinese University of Hong Kong, Hong Kong 999077, China

**Keywords:** TGF-β signaling, β cell, diabetes, proliferation, apoptosis, dedifferentiation, function

## Abstract

Beta (β) cell dysfunction or loss is the common pathological feature in all types of diabetes mellitus (diabetes). Resolving the underlying mechanism may facilitate the treatment of diabetes by preserving the β cell population and function. It is known that TGF-β signaling plays diverse roles in β cell development, function, proliferation, apoptosis, and dedifferentiation. Inhibition of TGF-β signaling expands β cell lineage in the development. However, deletion of *Tgfbr1* has no influence on insulin demand-induced but abolishes inflammation-induced β cell proliferation. Among canonical TGF-β signaling, Smad3 but not Smad2 is the predominant repressor of β cell proliferation in response to systemic insulin demand. Deletion of *Smad3* simultaneously improves β cell function, apoptosis, and systemic insulin resistance with the consequence of eliminated overt diabetes in diabetic mouse models, revealing Smad3 as a key mediator and ideal therapeutic target for type-2 diabetes. However, Smad7 shows controversial effects on β cell proliferation and glucose homeostasis in animal studies. On the other hand, overexpression of *Tgfb1* prevents β cells from autoimmune destruction without influence on β cell function. All these findings reveal the diverse regulatory roles of TGF-β signaling in β cell biology.

## 1. Introduction

Diabetes mellitus (diabetes thereafter) has become a global health issue with about 422 million victims in 2014 and 1.5 million-associated deaths in 2019 according to the report from World Health Organization [1]. Diabetes is a quite heterogeneous group of disorders that can be roughly classified into four subtypes: type-1 diabetes mellitus (T1DM), type-2 diabetes mellitus (T2DM), gestational diabetes, and other types like the maturity-onset diabetes of the young (MODY) [2]. T1DM, accounting for about 10% of the population with diabetes, is caused by the specific autoimmune attack against β cells in the islet, resulting in β cell destruction and absolute insulin deficiency [3]. T2DM, the major type of diabetes, has a more complex etiology with insulin resistance in peripheral tissues and β cell dysfunction or/and loss as the core mechanisms [3]. Gestational diabetes is defined as the “onset or recognition of diabetes or impaired glucose tolerance occurred during pregnancy” [4]. There is consensus that women suffering from gestational diabetes have a significantly higher risk to develop T2DM in the future [5]. The other types of diabetes have a relatively clear etiology usually associated with validated mutations of the specific genes [2].

Islet β cell is the only cell type to synthesize and secrete insulin to lower the blood glucose in adults [6]. β cell dysfunction or loss participates in the pathogenesis of almost all types of diabetes [7]. The central role of β cell in diabetes can be perceived by the fact that the majority of the identified single-gene mutations leading to diabetes, such as mutations in *PAX 6* [8], *HNF-1a*, *HNF-4a* [9], *GCK* [10], *PDX1* (*Ipf1*), etc. [11]), are involved with certain aspects of β cell biology including development, function, differentiation, proliferation, and apoptosis. Even in T2DM with complex etiology, most of the associated genetic factors are also related to the regulation of the compensatory function of β cells but not insulin resistance [12], therefore, highlighting the important role of β cells in the pathogenesis of diabetes.

There are many factors associated with β cell dysfunction or loss in diabetes. Genetic mutation-derived intrinsic defects and extrinsic metabolic stress (overwhelming systemic insulin demand) or other detrimental factors (like autoimmune antibodies, glucolipotoxicity, and inflammation) can independently or synergistically contribute to β cell dysfunction or loss. In these procedures, β cells may experience abnormal development, apoptosis, insufficient proliferation, dedifferentiation, and dysfunction with the consequence of overt diabetes [6,7,12,13,14,15]. All these biological processes, either in physiological or pathological conditions, are tightly modulated by various signaling pathways, including the TGF-β signaling [16].

TGF-β signaling is the prototype of TGF-β superfamily signaling, which also contains bone morphogenic protein (BMP), activin, Nodal, and growth and differentiation factor (GDF) signaling [17]. TGF-β signaling plays pleiotropic roles in various biological processes including cell growth and differentiation, development, apoptosis, cancer, fibrosis, immunity, and so on [18,19,20,21,22]. Accumulating evidence suggests that TGF-β signaling plays diverse roles in the development, proliferation, apoptosis, dedifferentiation, and function of islet β cells [16]. In this review, the new advances of TGF-β signaling in β cell biology and diabetes will be discussed. The potential application of β cell-based treatment of diabetes by targeting TGF-β signaling is also evaluated.

## 2. Overview of TGF-β Signaling

**Figure 1:**
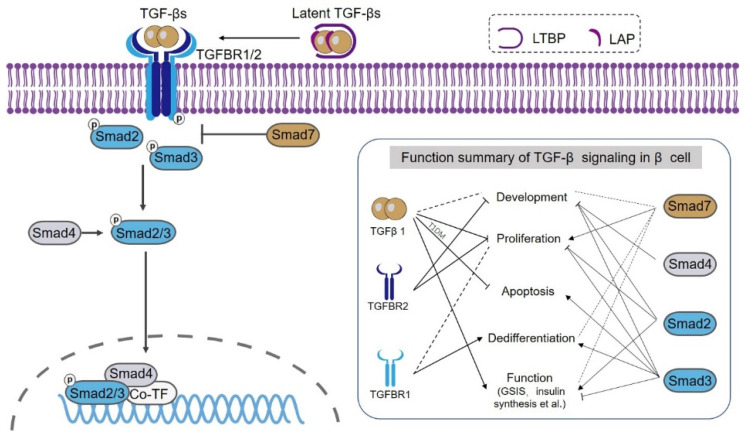
This schematic graph illustrates the classical TGF-β signaling and its roles in the regulation of β cell development, proliferation, apoptosis, dedifferentiation, and function. The TGF-β ligands are synthesized as latent TGF-βs. After being released, TGF-β binds to TGFBR2, which recruits and activates TGFBR1. TGFBR1 phosphorylates intracellular Smad2/3 which then binds to Smad4 and translocates into the nucleus to regulate the transcription of target genes. Smad7 negatively regulates TGF-β signaling by competing for the TGFBR1 with Smad2/3 and inducing the degradation of TGFBR1. The roles of each component of TGF-β signaling on different β cell biological processes are indicated in the insert. The solid line indicates a confirmative role while the dotted line suggests a role under debate. The line with the arrow represents positive regulation, while the line with blunted end stands for negative regulation. LAP, latency-associated peptide. LABP, latent TGF-β-binding protein.

TGF-β signaling has three ligands: TGF-β1, 2, and 3 which are universally expressed in various tissues and cell types. Among them, TGF-β1 is the most abundant ligand [19]. After synthesis, the mature TGF-β ligand is bound by latency-associated peptide (LAP) and latent TGF-β-binding proteins (LTBPs) to form the latent TGF-β with sequestered activity [17]. Latent TGF-β can be released and activated by specific enzymes or microenvironment factors, like plasmin, thrombospondin, integrin αvβ6, reactive oxygen species (ROS), and acid microenvironment [19,23,24]. For the canonical TGF-β signal transduction, the active TGF-β ligand is bound by the transmembrane receptor TGFBR2, resulting in the recruitment and activation of TGFBR1 by phosphorylation. Then, TGFBR1 phosphorylates the cytoplasmic receptor-regulated Smads (R-Smads) including Smad2 and Smad3. Phosphorylated Smad2/3 (p-Smad2/3) have transcriptional activity and can translocate into the nucleus after binding with Smad4 (common Smad, co-Smad) to regulate the transcription of target genes [17,19]. In addition, the TGF-β signaling also contains an inhibitory Smad protein Smad7 to counterbalance the signal strength by a negative feedback mechanism. Smad7 functions by binding to TGFBR1 to competitively block R-Smads phosphorylation [25]. Smad7 also promotes the degradation of TGFBR1 by inducing its ubiquitination mediated by E3 ubiquitin ligases such as Smurf2 [26]. The binding of R-Smad to the cis-regulatory element of target genes is also facilitated by additional partner transcriptional factors, thus together determining the specificity of transcriptional regulation in a context-dependent way [17] (Figure 1).

There also exist two additional auxiliary TGF-β coreceptors, betaglycan (also called TGFBR3) and endoglin (also called CD105), both of which are transmembrane glycoproteins [17]. Betaglycan has a widespread expression while endoglin is restricted mainly in vascular endothelia and particular hematopoietic lineages [27,28]. Betaglycan and endoglin function by binding to TGF-β ligand and facilitates its downstream binding to TGFBR1/2 [17]. The coreceptor can also be shed off as a soluble protein. Although the membrane-bound coreceptor assists the signaling activation, the soluble coreceptor suppresses the signaling activation by sequestering the TGF-β ligand [17].

In addition, the binding of the TGF-β ligand to the receptor also activates several non-Smad signaling to synergize the above canonical Smad signaling or function independently [29]. These non-Smad signaling include MAPK, PI3K/Akt, RhoA/ROCK, and IKK/NF-κB pathways (for a detailed review, see [29]). As there is currently no data on the role of non-Smad signaling in β cell biology, this review mainly focuses on canonical Smad signaling.

In the early embryonic development of pancreatic epithelia, TGFBR1/2, Smad2/3, and Smad7 are intensively expressed [30]. The expression of TGFBR1/2 and Smad2/3 persists till the late developmental stage of endocrine lineage but is gradually restricted in postnatal islets, while Smad7 turns out to be barely detected [30,31,32]. Furthermore, TGF-β1 also shows diffuse expression in the islet of both humans and rodents [32,33]. These findings suggest that TGF-β signaling keeps a considerable activity in the developing and mature islet cells under physiological conditions. In situations of metabolic syndrome and diabetes, TGF-β signaling is systemically activated as the serum levels of TGF-β1 are elevated in patients with obesity and diabetic animal models along with increased levels of p-Smad2/3 in various tissues [34,35,36]. Increased nuclear translocation of Smad3 or p-Smad3 is also observed in islets of diabetic mice [37,38]. Functional investigations further uncovered the diverse roles of the different components of TGF-β signaling in β cell biology and their tight association with diabetes (Table 1).

## 3. TGF-β Ligand

In embryonic organogenesis, the pancreas is derived from the endodermal foregut [60]. From embryonic day 9.0 (E9.0) to 12.5 in mice, the pancreatic bud gradually emerges as the evagination of foregut endoderm to form the dorsal and ventral buds wherein the stratified pancreatic progenitor cells form a duct structure with a centralized lumen. Later on, progenitor cells localized in the tip of the pancreatic duct will differentiate into acinar cells, while those localized in the stalk will give rise to endocrine cells (mainly α, β, δ, and PP cells) [60,61]. Previous studies reported the controversial roles of TGF-β ligand in the fate determination of pancreatic progenitor cells into endocrine (islet) cells and exocrine acini. An early study in cultured mouse embryonic pancreatic rudiments (E12.5) found that TGF-β1 can suppress the acinar but promote endocrine tissue differentiation [39]. However, a later study showed that the addition of neither TGF-β1 nor its neutralizing antibody influences the endocrine cell differentiation and β cell mass in cultured pancreatic rudiment, although the islet structure is significantly perturbed due to inhibition or promotion of cell migration [44]. It is also reported that antibody-based blockade of TGF-β signaling can promote the endocrine β cell differentiation in ex vivo pancreatic rudiment, indicating an inhibitory role of TGF-β on endocrine development [31].

Despite these controversial findings of TGF-β in β cell differentiation in cultured pancreatic rudiment, mice with β cell-specific overexpression of *Tgfb1* under the rat *Ins2* promoter show progressive fibrosis in the acinar tissue around the islet without alterations in β cell development, β cell mass, and insulin secretion [43]. Furthermore, these transgenic mice maintain normal blood glucose [43]. A similar phenomenon is also observed in an independent mouse model with transgenic expression of *Tgfb1* in β cells driven by human insulin promoter [42]. As insulin initiates its expression early during the endocrine differentiation, these data from transgenic animals suggest that TGF-β1 may not influence the endocrine differentiation in pancreatic development and postnatal β cell mass, although overexpression of *Tgfb1* causes fibrosis in acinar tissue [42,43].

Another interesting finding is that β cell-specific overexpression of *Tgfb1* in non-obese diabetic (NOD) mice, an autoimmune T1DM model, can protect the β cells from autoimmune damage resulting in normalized blood glucose, although these mice developed much smaller pancreas with massive fibrosis [46]. Similar protective effects against β cell autoimmune injury and diabetes were also observed in NOD mice with α cell-specific *Tgfb1* overexpression [45]. These findings keep in line with the immune-suppressive activity of TGF-β1 as reported in *Tgfb1*-null mice and other autoimmune diseases [62,63].

TGF-β1 also shows diverse roles on β cell proliferation and insulin secretion. In rat islets cultured in a low glucose condition (11.1 mM), TGF-β1 treatment promotes insulin secretion without influencing cell replication. However, under the high glucose condition (16.7 mM), TGF-β1 has no effect on insulin secretion but suppresses high glucose-stimulated cell replication [40]. Nevertheless, this finding is challenged by a later study showing that TGF-β1 treatment of mouse islets suppresses the expression of genes involved with insulin synthesis (including *Ins1* and *Ins2*), processing, and secretion [41].

## 4. TGFBRs

At the embryonic stage, TGFBR1 and 2 show parallel expression in pancreatic epithelium and also weak expression in the mesenchyme (E11.5 and E12.5). Later on (E14.5 afterward), their expression is restricted to the cord region of the pancreatic duct which is committed for endocrine lineage differentiation [31]. Overexpression of dominant-negative *Tgfbr2* under the metallothionein 1 (*Mt-1*) promoter favors the embryonic development of endocrine lineage by enhancing proliferation. However, the adult transgenic mice show normal endocrine phenotype which may be associated with the change of expression of dominant-negative TGFBR2 from endocrine progenitor (cord region of the pancreatic duct) at the embryonic stage to the acini in adult animals [31,64]. Consistently, the adult transgenic mice demonstrate ectopic proliferation and perturbed differentiation of acinar cells with massive fibrosis in the exocrine tissue [64]. However, the increased expansion of endocrine lineage in embryonic development seems to have little influence on adult β cell mass as mice lacking *Tgfbr2* alone or in combination with *Tgfbr1* early in pancreatic progenitor cells (Cre driven by *Ptf1a* promoter, *Ptf1a*-Cre) demonstrate normal adult β cell mass, blood glucose level, and glucose tolerance [32,53].

β cell mass expansion can be achieved by replication of pre-existing β cells, neogenesis from progenitor cells, and transdifferentiation from other cell types (like α cell) [65,66]. It is now assumed that the expansion of β cell mass in the physiological conditions and in response to metabolic stress/systemic insulin demand occurs mainly through self-replication of the pre-existing β cell [67,68], which is partially regulated by TGF-β signaling. In islets of normal mice, TGFBR2 is massively activated (p-TGFBR2) but soon inactivated in the early 24 h upon partial pancreatectomy (60%) [32]. Knockout of *Tgfbr2* in pancreatic progenitor cells (driven by *Ptf1a*-Cre) or in adult β cells (tamoxifen-inducible Cre driven by *Pdx1* promoter, *Pdx1*-Cre-ERT) significantly promotes the β cell proliferation in mice with partial pancreatectomy [32]. Although TGFBR1 is as important as TGFBR2 to relay TGF-β signaling, deletion of *Tgfbr1* with *Ptf1a*-Cre or *Pdx1*-Cre-ERT has no influence on β cell proliferation in the same mouse model of partial pancreatectomy [32]. This is contrary to other studies showing that pharmacological inhibition of TGFBR1 by chemical inhibitors (like SB431542, SB505124, and SD208) promotes the proliferation of both mouse and human β cells in vitro and in vivo (in allograft for human β cells) [49,50,51].

In addition to systemic insulin demand, it has been reported that pancreatic inflammation or pancreatitis also induces β cell proliferation wherein TGFBRs seem to play distinct roles. Xiao et al. reported that deletion of both *Tgfbr1* and *2* suppresses inflammation-induced β cell proliferation in mice with pancreatic duct ligation-induced inflammation [53]. Mechanistic analysis found that in inflammation-induced β cell proliferation, M2 macrophage plays a pivotal role in TGF-β1 secretion to upregulate the pro-proliferative Smad7 which is mediated by TGFBRs. M2 macrophage also releases epidermal growth factor (EGF) to block the activation of anti-proliferative Smad2 (the roles of Smad2 and 7 will be discussed later on) [52]. Consistently, pharmacological inhibition of TGFBR1 by SB431542 abrogates the pro-proliferative effect rendered by M2 macrophage on cocultured β cells [52]. Therefore, TGFBR1 shows disease-dependent roles in the regulation of β cell proliferation.

β cell dedifferentiation is defined as the loss or decreased expression of marker genes (like insulin, *Glut2*, *Mafa*, and *Pdx1*) of mature β cells while shifting to a progenitor status (positive for Neurog3) with abolished or reduced insulin secretion upon glucose stimulation [69]. It is reported that β cell dedifferentiation but not apoptosis is the major cause of decreased functional β cell pool in the animal models of T2DM [70]. β cell dedifferentiation is also confirmed in pancreas autopsy of patients with T2DM and can also be observed in cultured islet β cells [69,71,72]. Toren-Haritan and Shimon Efrat reported that long-term culture of human β cells leads to marked dedifferentiation, with the loss of β cell-specific transcripts and activation of TGF-β signaling. However, shRNA-mediated knockdown of TGFBR1 blocks the dedifferentiation of cultured human β cells [47]. Mechanistically, TGFBR1 knockdown can activate AKT/FOXO1 pathway to upregulate *Neurod1* and *Mafa*, both of which are important transcriptional factors to maintain insulin expression [47]. Surprisingly, knockdown of TGFBR1 decreases the proliferation of these long-term cultured human β cells with increased expression of cell cycle inhibitors p21 and p27 [47], which is contrary to the findings in vivo and in short-term cultured human β cells [49,51].

The coreceptor endoglin closely relates to angiogenesis and endothelial function [54]. Mice null of endoglin die during embryonic development because of cardiovascular defects [73]. Patients with diabetes show increased plasma levels of soluble endoglin [74]. Furthermore, the high-fat diet (HFD)-fed mice with heterozygous deletion of endoglin demonstrate decreased serum insulin but with normal body weight, blood glucose, and insulin sensitivity [75]. These data suggest a possible role of endoglin in islet physiology. In islet, endoglin is detected in endothelia and also in mesenchymal stromal cells [76]. In an in vitro coculture assay, the islet-derived endoglin-expressing stromal cells can cause impaired viability and function of the endothelial cells while the exact role of endoglin in this process remains elusive [76]. On the other hand, mice with inducible deletion of endoglin in endothelial cells have a normal islet phenotype despite a lower density of intra-islet blood vessels [54]. As intra-islet blood vessel is important for glucose sensing and insulin distribution [77], whether endothelia-specific endoglin would play a role in islet function in stressed conditions, such as diabetes, could be an interesting issue. Unlike endoglin, there are limited investigations on the role of the other TGF-β coreceptor betaglycan in β cells.

## 5. Smad2/3

As shown in Figure 1, although both Smad2 and 3 are downstream signal transducers of TGF-β signaling, they regulate distinct target genes and execute different biological roles [78]. For example, in chronic kidney disease, Smad3 promotes but Smad2 inhibits renal fibrosis [20]. In addition, *Smad2*-null mice are embryonic lethal caused by severe developmental defects while *Smad3*-null mice are viable despite mild bone malformation and immune dysfunction [79,80,81,82]. Smad2 and 3 have the same DNA binding motif of “CAGA” [78]. However, this four-nucleotide motif implies low affinity and specificity of DNA binding by Smad2/3. Therefore, additional binding partners/transcriptional factors are required to synergize the efficient binding of Smad2/3 to the cis-regulatory element, which largely dictates the selection of target gene and regulatory fashion (transcriptional priming or repression) [83].

Although it is reported that heterogenous *Smad2*-null mice develop islet hypoplasia [55], mice with β cell-specific deletion of *Smad2* (under the rat insulin promoter (RIP)-driven Cre, RIP-Cre) show enhanced expansion of β cell mass with normal β cell lineage differentiation [30,32,56]. Although *Smad3*-null mice show unchanged β cell mass after birth, an increased β cell proliferation is also observed during embryonic development [30,32,41]. These data suggest that Smad2 and 3 are suppressive in β cell development but not essential for its specification. However, Smad2 and 3 demonstrate different roles in β cell proliferation and function postnatally.

Similar to TGFBR2, p-Smad2/3 is robustly detected under physiological conditions in adult mouse islets [32]. However, in mice with partial pancreatectomy, the residual islets show decreased levels of p-Smad2/3 accompanied by enhanced β cell proliferation. Further genetic analysis found that the deletion of *Smad3* (*Smad3*-null) results in much more robust β cell proliferation than the deletion of *Smad2* (under *Pdx*-Cre-ERT or *Ptf1a*-Cre) [32]. On the other hand, the double deletion of *Smad2* and *3* has no additive effect on β cell proliferation compared with sole *Smad3*-deleted mice [32]. These findings suggest that Smad3 but not Smad2 is the predominant downstream transcriptional factor of TGF-β signaling to suppress β cell proliferation. Most importantly, our recent work showed the deletion of *Smad3* completely prevents overt diabetes in db/db mice. These *Smad3*-deficient db/db mice (Smad3KO-db/db) demonstrate β cell hyperplasia, persistent hyperinsulinemia, normal glycemia, recovered glucose tolerance, and eliminated insulin resistance [37]. Increased β cell proliferation is also observed in cultured *Smad3* knockout islet cells [84]. Furthermore, db/db mice also present massive β cell dedifferentiation with decreased expression of characteristic genes of mature β cells, like *Pax6*, *Pdx1*, *Mafa*, *Nkx6.1*, *Neurod1* et al. the expression of which, however, are recovered in Smad3KO-db/db mice [37].

β cells can undergo apoptosis as a consequence of unsolvable workload-induced ER stress and glucolipotoxicity in T2DM [6,85]. β cell apoptosis, although happening in low incidence, has been well documented in diabetic animal models and pancreatic autopsy in patients with T2DM [38,86,87]. Smad3 also plays a pivotal role in the regulation of β cell apoptosis in the conditions of diabetes or metabolic syndrome. Islets from HFD-fed mice show increased β cell apoptosis, which is accompanied by the activation of Smad3 (p-Smad3) [38]. Doxycycline-inducible β cell-specific overexpression of dominant-negative *Smad3* (Smad3DN) prevents HFD-induced diabetes by decreasing β cell apoptosis, while overexpression of constitutively active *Smad3* (Smad3CA) exacerbates diabetes with an increase in β cell apoptosis [38]. Notably, overexpression of Smad3CA at the embryonic stage also enhances β cell apoptosis, resulting in decreased β cell mass in newborn mice [38].

Smad3 not only influences β cell proliferation and apoptosis but also regulates insulin synthesis and secretion (Figure 1). It is reported that TGF-β1 represses insulin gene transcription [41]. Furthermore, a functional study proved that Smad3 but not Smad2 executes the transcriptional repression effect on insulin [41]. Insulin is a *bona fide* target gene of Smad3 as Smad3 suppresses insulin transcription by directly binding to its promoter [41]. In cultured β cell line INS-1E, knockdown of Smad3 increases both the intracellular and secreted insulin [41]. Importantly, *Smad3*-deficient mice show a slightly higher level of blood insulin, pancreatic insulin content, and moderate hypoglycemia with augmented glucose tolerance, although these mice have similar islet morphology and β cell mass compared with the wild-type mice [41]. Furthermore, Smad3 deficiency not only enhances insulin synthesis but also promotes glucose-stimulated insulin secretion (GSIS) in vivo and in cultured islets accompanied by upregulated expression of genes involved with insulin biosynthesis, processing, GSIS, and β cell maturation [41].

In contrast to Smad3, mice with β cell-specific deletion of *Smad2* with RIP-Cre (Smad2βKO) show normal blood glucose but impaired glucose tolerance and GSIS [56]. Further analysis indicates a functional deficiency of the KATP channel, which is essential for GSIS, in *Smad2* knockout β cells [56]. These mice also develop age-dependent islet hyperplasia with increased β cell mass which may be caused by the adaptive β cell proliferation induced by insufficient insulin supply [56].

It has been well documented that TGF-β/Smad3 signaling induces cell cycle arrest mainly by promoting the expression of cyclin-dependent kinase inhibitors (CDKIs) in epithelial cells [21]. A similar mechanism also exists in β cells. Dhawan et al. reported that Smad3 suppresses adult β cell proliferation by inducing p16 expression [49]. Mechanistically, Smad3 directly interacts with histone methyltransferase Mll1 and facilitates the recruitment of trithorax complex to and epigenetically activates the *Ink4a/Arf* locus, which encodes p16, through promoting the trimethylation of lysine 4 in histone H3 (H4K4me3) [49]. In another study, Wang et al. demonstrated that TGF-β signaling induces p21, p15, and p57 to repress cell cycle in cultured human islets, which is also involved with trithorax complex-mediated epigenetic modification [51]. On the contrary, our recent data showed that *Smad3* deficiency has no influence on CKDIs expression in islets from young-aged mice both in vitro and in vivo [84]. Further analysis revealed that Smad3 represses the cell cycle in β cells by transcriptionally suppressing *E2f3*, a key gene promoting G1/S cell cycle entry [84]. Therefore, different molecular mechanisms mediate TGF-β signaling-regulated β cell proliferation in a context-dependent manner. There also exist many other signals functioning upstream of the cell cycle pathway, that show significant influence on β cell proliferation and are proven to be regulated by TGF-β signaling, like Akt/mTORC1, Wnt/β-catenin, and PKA/CREB [88,89]. It is likely that these signals may also be involved with TGF-β-regulated β cell proliferation.

## 6. Smad4

Smad4 is the binding partner of Smad2/3 and a well-characterized tumor repressor that is mutated in more than 50% pancreatic ductal carcinomas [90]. However, studies on the role of Smad4 in islet β cells are limited. An early study by Simeone et al. showed that suppression of Smad4 by transgenic overexpression of dominant-negative *Smad4* mutant under the elastase promoter causes an age-dependent increase of islet size without influencing the glucose homeostasis in normal feeding conditions. One thing that needs to note is that these transgenic mice show only mosaic expression of the mutated Smad4 in acinar cells and stromal cells without detectable expression in the islet. Further analysis reveals that Smad4 inhibition may promote β cell neogenesis by enhancing endocrine lineage differentiation from the pancreatic duct [57].

## 7. Smad7

Smad7 is expressed in the early developmental stage of pancreatic progenitor cells [30]. In the later gestational stage and adult pancreas of physiological conditions, Smad7 demonstrates minimal or absent expression in pancreatic islets [30,32]. In mice with partial pancreatectomy, Smad7 is quickly induced in islets in 24 h and presents non-overlapped distribution with insulin. Moreover, Smad7-positive cells manifest increased proliferation [91]. Interestingly, lineage tracing analysis found that these Smad7-positive but insulin-negative (Smad7+; insulin-) cells are originated from pre-existing β cells. Most of them co-express pancreatic polypeptide (PP), a hormone also expressed in endocrine progenitor cells [91], suggesting that β cells may undergo transient dedifferentiation during replication [32]. Most importantly, deletion of *Smad7* almost completely abrogates β cell proliferation induced by partial pancreatectomy, implying an essential role of Smad7 in the insulin demand-induced β cell proliferation [32]. Smad7 is also essential for basal β cell proliferation as its deletion suppresses β cell proliferation under physiological conditions [59].

In addition, Smad7 is also found to be essential for inflammation-induced β cell proliferation. Xiao et al. reported that in mice with pancreatic duct ligation, the infiltrated M2 macrophage secretes TGF-β1 to upregulate pro-proliferative Smad7 and EGF to suppress anti-proliferative Smad2 in β cells, resulting in increased β cell proliferation which is abolished by β cell-specific deletion of *Smad7* [52]. Importantly, β cell-specific overexpression of *Smad7* alone by intra-pancreatic ductal virus (adeno-associated virus, AAV) delivery can trigger increased β cell proliferation and β cell mass in mice without pancreatic duct ligation [52]. This suggests a cellular autonomous pro-proliferative role of Smad7 in β cell.

Using a doxycycline-inducible Tet-Off transgenic strategy, Smart et al. showed that embryonic overexpression of *Smad7* (Driven by *Pdx1* promoter) results in abnormal development of the pancreas, spleen, and duodenum. These transgenic mice die at day one postnatally with an 85–90% reduction of β cells and increased α cells. On the other hand, inducible overexpression of *Smad7* in adult mice causes overt diabetes with hypoinsulinemia, impaired glucose tolerance, and reduced pancreatic insulin content [58]. Importantly, the diabetic phenotype can be reversed upon cessation of the transgene expression [58]. Unlike at embryonic stage, *Smad7* overexpression in adult β cell causes mild islet hyperplasia with decreased levels of Menin and p27 [58], two cell cycle inhibitors that repress β cell proliferation [92,93,94], while reducing the expression of Mafa, a key transcriptional factor to maintain insulin expression [95]. However, *Smad7* overexpression does not reduce the levels of Nkx6.1 and Neurod1, two other markers of mature β cell [58]. Overt diabetes is also observed in adult mice with conditional *Smad7* overexpression under the RIP promoter using the doxycycline-inducible Tet-On system [58]. In contrast to the above findings by Smart, a recent study from George Gittes’ group showed that *Smad7* overexpression in adult β cells promotes β cell proliferation without significant alteration in β cell mass and glucose homeostasis [59]. Unlike Smart’s results, they found that *Smad7* overexpression induces dedifferentiation in β cells as revealed by decreased expression of Pdx1, Mafa, and Nkx6.1 [59]. Therefore, based on the current findings, a pro-proliferative role of Smad7 in adult β cells is assumed (Figure 1). However, more evidence is warranted to clarify the exact role of Smad7 in β cell function and diabetes.

## 8. Other Possible Mechanisms Involved with TGF-β Signaling-Regulated β Cell Dysfunction and Loss

TGF-β signaling initiates many cellular processes which have been independently validated to participate in β cell dysfunction and loss in diabetic conditions. Among them, chronic inflammation and oxidative stress are the common characters of diabetes, especially for T2DM, which happens not only in peripheral tissues but also in islets [6,96]. Inflammation and oxidative stress can be provoked by glucolipotoxicity and workload-induced endoplasmic reticulum stress (ER stress), and mediate the deterioration of β cell function and survival [85,96]. Furthermore, oxidative stress can induce inflammation and vice versa [15,97]. In islets, inflammatory cytokines can be produced by the infiltrated immune cells (like macrophage) and β cells themselves [96,98].

TGF-β signaling plays diverse roles in the regulation of inflammation [99]. As described above, TGF-β signaling is required to keep the immune quiescence of T cells in the physiological conditions as mice with *Tgfb1* knockout or T cell-specific deletion of *Tgfbr2* develop multi-organ autoimmune injury [63,100,101]. Accordingly, overexpression of *Tgfb1* improves autoimmune β cell injury in the mouse model of T1DM [45,46]. Furthermore, abnormal immunity, although less severe, was also observed in *Smad3*-null mice, which is also involved with dysregulated T cell activation [80]. In contrast to the suppressive role of TGF-β signaling on T cell-mediated adaptive immunity, the downstream effectors Smad2 and 3 promote innate immunity-associated inflammation in other organs (see review references [20,99,102]). In respect of T2DM, deletion of *Smad3* attenuates inflammation in kidney and heart of db/db mice [36,103]. However, evidence of the role of TGF-β signaling-regulated inflammation in β cells in T2DM is lacking.

β cell undergoes robust synthesis of insulin with the production of excessive ROS [6]. However, β cell has a relatively lower antioxidative ability, making it more sensitive to oxidative stress and associated dysfunction [6,104]. TGF-β1 can induce oxidative stress by promoting ROS production from mitochondria and NADPH oxidase 4 (Nox4) [105]. Mechanistically, TGF-β1 increases ROS by inducing the mitochondrial respiratory defect by inhibiting the activity of complex IV through deactivating (phosphorylating) GSK3 α and β in lung epithelial cells [106]. In the case of Nox4-derived ROS, TGF-β1 promotes *Nox4* expression in both Smad-dependent (mainly by Smad3) and independent (via PI3K, MAPK, and RhoA/ROCK) pathways [105]. On the other hand, TGF-β1 also reduces the intracellular antioxidants including glutathione (GSH), superoxide dismutase (SOD), catalase, and glutaredoxin (Grx) [105]. Despite the above progresses, whether oxidative stress mediates the regulatory role of TGF-β signaling in β cell dysfunction remains unclear.

Autophagy is an important cellular behavior to maintain intracellular homeostasis by degrading organelles and proteins for recycling purposes [107]. Autophagy is also responsible to remove the defective organelles and proteins which happens especially in β cells under diabetic stress [107]. Mice with β cell-specific deficiency of autophagy show degenerated β cells, hyperglycemia, reduced insulin release, and impaired glucose tolerance. Moreover, these abnormalities are exacerbated in the condition of HFD feeding, suggesting that autophagy is required for normal β cell survival and function in both physiological and diabetic conditions [108]. TGF-β is able to activate autophagy in various cell types through the Smad and non-Smad signaling, which might be an adaptive response [109]. Currently, in diabetic nephropathy, Smad3 was found to repress TFEB-dependent lysosome biogenesis by transcriptionally suppressing *TFEB* expression, resulting in autophagy dysregulation in renal epithelial tubular cells [110]. It would be interesting to investigate whether TGF-β signaling regulates β cell dysfunction and loss through modulating autophagy.

There exist some small metabolites which also play important roles in β cell biology. One example is 2-methoxyestradiol (2ME), a metabolic derivative of estradiol that shows estrogen receptor-independent anticancer activity [111]. Administration of 2ME in db/db mice promotes β cell expansion, elevates serum insulin, lowers blood glucose, and improves glucose intolerance but without influence on insulin resistance [112]. 2ME also suppresses the TGF-β3-induced phosphorylation of Smad2/3 and fibrosis in immortalized human uterine fibroid smooth muscle (huLM) cells and in rats with benign prostate hyperplasia [113,114]. However, whether the suppression of TGF-β signaling by 2ME is also involved with its beneficial role in β cells remains unclear.

## 9. β Cell-Based Treatment of Diabetes by Targeting TGF-β Signaling

β cell dysfunction or loss participates in the pathogenesis of all types of diabetes. Considering the important and diverse regulatory roles of TGF-β signaling in the development, differentiation, biological function, apoptosis, and dedifferentiation of β cells, it is reasonable to explore the potential anti-diabetic treatment by targeting the TGF-β signaling.

It is reported that pan-TGF-β blockade by antibody ameliorates diabetes by improving obesity, glucose intolerance, insulin resistance, and hyperglycemia in the type-2 diabetic db/db and HFD-fed mice [34]. However, it is not clear whether these improvements rendered by TGF-β blockade also benefit the β cell survival and function. There also remains a concern for the anti-TGF-β treatment in diabetes as TGF-β1 has immunosuppressive activity [62,63]. The long-term anti-TGF-β1 treatment may result in impaired immunity with an increased risk of infection. Nevertheless, the immunosuppressive activity of TGF-β1 also cast therapeutic potential for autoimmune T1DM as islet-specific *Tgfb1* overexpression abolishes anti-β cell autoimmune response, resulting in regression of the diabetic phenotype in NOD mice [45,46]. Therefore, islet/β cell-targeted delivery of TGF-β1 may be promising in the treatment of T1DM.

Genetic deletion of *Tgfbr2* promotes β cell proliferation in response to insulin demand [32,53]. Although *Tgfbr1* deficiency has no influence on insulin demand-induced β cell proliferation [32], its pharmacological inhibition elevates β cell proliferation in vitro and in vivo in both rodents and humans [49,50,51]. In addition, dedifferentiation can also be reversed by targeted inhibition of TGFBR1 in cultured β cells [48]. However, there is a lack of in vivo evidence to show whether TGFBR deficiency or suppression can ameliorate diabetes. For the downstream Smad proteins, Smad2 is proven to be essential for GSIS [56], making it inappropriate for pharmacological intervention. The importance of Smad2 in β cell function also implies that targeted inhibition of TGFBRs may not be the best option in the treatment application. Although overexpression of Smad7 shows a consistent proliferation-promoting effect on β cells in adult mice, it has controversial roles on β cell function, which need to address with more studies [58,59]. Smad4 is a tumor repressor whose mutation is highly associated with pancreatic ductal carcinomas, which excludes it as a therapeutic target [90].

**Figure 2:**
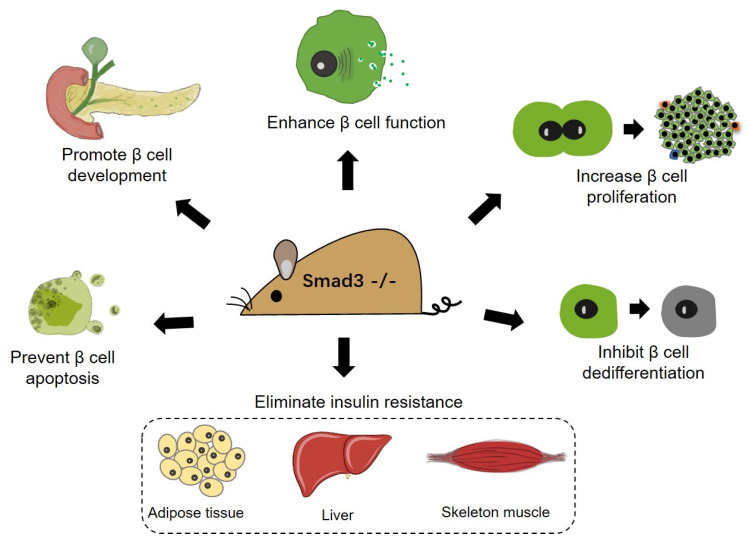
Benefits of *Smad3* deficiency on β cell biology and insulin resistance. *Smad3* deficiency in mice promotes β cell development, augments β cell function (by elevating insulin synthesis and GSIS), enhances β cell proliferation in response to systemic insulin demand, and suppresses/eliminates β cell apoptosis, β cell dedifferentiation, and insulin resistance in conditions of T2DM.

Accumulating evidence supports the promising application of Smad3-targeted treatment of diabetes. Smad3 is not essential for normal β cell development, but its deletion promotes β cell function by improving insulin synthesis, processing, and secretion [41]. In physiological conditions, *Smad3*-null mice show normal β cell mass suggesting no significant or modest, if any, promotion of β cell proliferation [41]. However, in response to metabolic stress (systemic insulin demand), robustly enhanced β cell proliferation can be observed in *Smad3*-deficient mice [32]. Our study also demonstrated that *Smad3* deletion prevents overt diabetes in db/db mice by promoting islet hyperplasia and hyperinsulinemia accompanied by eliminated β cell dedifferentiation [37], suggesting that enhanced β cell mass and function may contribute to the amelioration of T2DM in *Smad3* knockout db/db mice (Figure 2). Next, this notion was validated in *Smad3*-deficient islet transplantation in db/db mice in our recent work [84]. Furthermore, *Smad3* deficiency also eliminates insulin resistance, the most important causal factor of T2DM, in peripheral organs like liver, skeleton muscle, and adipose in HFD-fed mice and db/db mice [34,37]. In HFD-fed mice, *Smad3* deficiency facilitates the white adipose tissue acquiring a brown adipose tissue-like phenotype with increased mitochondria biogenesis and energy expenditure by promoting PGC-1α expression, resulting in the resolution of insulin resistance and overt diabetes [34]. These exciting findings suggest that Smad3 could be a suitable target for systemic intervention for the treatment of T2DM (Figure 2). Further, in islet transplantation therapy of T1DM, the enrollment of *Smad3*-deficient islets largely lowers the number of transplanted islets required to recover euglycemia in streptozocin (STZ)-induced diabetic mice, which is associated with increased β cell proliferation in islet graft [84].

## 10. Conclusions, Perspectives, and Future Directions

Based on the available findings, TGF-β signaling is activated in the developing and adult β cells and plays a generally suppressive role on β cell proliferation. Nevertheless, there remain many questions to be addressed. One major concern is the paradoxical roles of TGFBR1 whose deletion shows no influence on insulin demand-induced but abolishes inflammation-induced β cell proliferation. Both overexpression and deletion of *Smad7* suppress embryonic β cell development, indicating a precise *Smad7* expression is pivotal for normal β cell development. Although Smad7 presents pro-proliferative activity in adult β cells, its roles on β cell function and glucose homeostasis remain controversial. Inhibition of TGFBR1 induces the redifferentiation of dedifferentiated β cells in vitro. It is not clear whether inhibition of TGFBR1 or the other components of TGF-β signaling has the same effect in vivo. Smad3 but not Smad2 is the primary repressor of β cell proliferation in response to workload. However, *Smad3* deficiency promotes but *Smad2* deficiency impairs β cell function. Resolution of the partner factors synergizing the DNA binding and selection of the target genes would help to understand the distinct roles of Smad2 and 3 in β cell biology.

From the therapeutic perspective, local delivery of TGF-β1 is promising to treat T1DM by protecting the β cells from autoimmune attack. The most exciting finding is the pathogenic role of Smad3 in T2DM. *Smad3* deficiency facilitates insulin demand-induced β cell proliferation and function, prevents stress-induced β cell apoptosis and dedifferentiation, and eliminates peripheral insulin resistance, thus resulting in the regression of overt diabetes in *Smad3* knockout mouse models of T2DM (both db/db and HFD-fed mice). Therefore, exploration of Smad3-targeted expression modification or chemical inhibitors may be promising approaches for the treatment of T2DM. Recently, it is reported that administration of SIS3, a specific inhibitor of Smad3, in HFD-fed mice reduces body weight and hyperglycemia with improved glucose intolerance and insulin resistance [115]. Our unpublished data also suggests a protective role of SIS3 against diabetic phenotype with improved β cell survival in db/db mice. An alternative approach is to silence Smad3 by antisense oligonucleotides (ASOs), which can be delivered systemically, or specifically to β cells upon conjugation to the ligand of glucagon-like peptide-1 receptor (GLP1R) [116]. Moreover, the rapidly-developed CRISPR-Cas13 RNA modification technology can silence the pathogenic gene more efficiently and specifically than RNA interference without perturbing the DNA permanently [117,118], which would be a valuable tool to target Smad3 in future treatment of β cell dysfunction/loss and T2DM.

For future directions, a deeper investigation of the molecular mechanism underlying the diverse functions of TGF-β signaling in β cell biology is warranted for the better treatment of β cell-related disorders by targeting this signaling. These include: (1) more well-designed studies are needed to address the paradoxical roles of TGFBR1 and Smad7 in environment-dependent β cell biology; (2) to uncover the partner factors synergizing Smad2/3 for the regulation of distinct target genes, which may help to explain the distinct roles between Smad2 and 3 in β cell biology; (3) to explore the role of non-Smad signaling in β cell biology, which is still an attractive virgin land; (4) despite the encouraging findings of *Smad3* deficiency in the improvement of β cell disorder and T2DM, and the beneficial role of TGF-β1 against autoimmune insults to β cells in T1DM, the efficient therapeutic methodologies remain to develop.

## Figures and Tables

**Table 1:** Role of different components of TGF-β signaling in β cell biology.

Component	Biological Function in β Cell/Diabetes	Experimental Model and Parameter	Reference
TGF-β ligand	Promote/suppress β cell development.	Cultured pancreatic rudiment treated with TGF-β1.	[31,39]
	Promote insulin secretion in low glucose condition (11.1 mM).	Cultured rat islets treated with TGF-β1.	[40]
	Suppress transcription of genes related to insulin synthesis, processing, GSIS, and β cell maturation.	Cultured mouse islets treated with TGF-β1.	[41]
	Disorganized (smaller) islet but with normal β cell development, β cell mass, and insulin secretion.	Mice with transgenic expression of *Tgfb1* in β cell.	[42,43]
	No influence on β cell development but with perturbed islet structure.	Cultured rat pancreatic rudiment treated with TGF-β1 or its neutralizing antibody.	[44]
	Protect against autoimmune T1DM.	NOD mice with *Tgfb1* overexpression in β or α cell.	[45,46]
TGFBR1	Promote redifferentiation but suppress proliferation of dedifferentiated β cell upon knockdown or pharmacological inhibition.	Long-termed (3 weeks) culture of human and rodent islet cells.	[47,48]
	Promote β cell proliferation upon pharmacological inhibition.	Cultured mouse and human β cell; mice treated with chemical inhibitor; human islet allograft.	[49,50,51]
	Suppress β cell proliferation upon pharmacological inhibition or combinational deletion with *Tgfbr2*.	Mice with pancreatic duct ligation; β cell cocultured with M2 macrophage.	[52,53]
	No influence on β cell proliferation.	Mice with partial pancreatectomy and conditional knockout under *Pdx1*-Cre-ERT or *Ptf1a*-Cre.	[32]
TGFBR2	Promote β cell proliferation upon deletion.	Mice with partial pancreatectomy and conditional knockout under *Pdx1*-Cre-ERT or *Ptf1a*-Cre.	[32]
	Suppress β cell proliferation upon combinational deletion with *Tgfbr1*.	Mice with pancreatic duct ligation.	[53]
endoglin	Suppress the formation of intra-islet blood vessels upon deficiency.	Mice with inducible deletion of endoglin in endothelial cells	[54]
Smad2	Islet hypoplasia upon heterogeneous deletion.	Heterogeneous *Smad2*-null mice.	[55]
	Promote β cell proliferation upon deletion.	Mice with partial pancreatectomy and conditional knockout under *Pdx1*-Cre-ERT or *Ptf1a*-Cre.	[32]
	Cause islet/β cell hyperplasia but β cell dysfunction with impaired insulin secretion and glucose homeostasis upon conditional deletion in β cell.	Mice with conditional *Smad2* knockout under RIP.	[56]
Smad3	Directly repression of insulin transcription by promoter binding; repress insulin synthesis and secretion; attenuate GSIS and glucose tolerance	β cell line INS-1E; *Smad3*-deficient mice and corresponding islets	[41]
	Promote β cell proliferation in response to systemic insulin demand upon deletion.	Mice with partial pancreatectomy; db/db mice.	[32,37]
	Promote β cell apoptosis.	HFD-fed mice	[38]
Smad4	Induce islet hyperplasia upon deletion.	Mice overexpressing dominant-negative Smad4 under elastase promoter.	[57]
Smad7	Suppress β cell lineage development upon overexpression at the gestational stage.	Doxycycline-inducible (Tet-Off) β cell-specific *Smad7* overexpression under the regulation of *Pdx*-1 promoter during gestation.	[58]
	Promote β cell proliferation in response to systemic insulin demand and local pancreatic inflammation, and also in physiological conditions.	Mice with partial pancreatectomy and conditional *Smad7* knockout under *Pdx1*-Cre-ERT or *Ptf1a*-Cre or *Ngn3*-Cre; mice with β cell-specific *Smad7* overexpression by AAV-mediated gene delivery; mice with pancreatic duct ligation; β cell-specific *Smad7* overexpression in adult mice.	[32,52,59]
	Involved with β cell dedifferentiation but does not influence β cell function and glucose homeostasis upon overexpression.	Doxycycline-inducible (Tet-On) β cell-specific *Smad7* overexpression under the regulation of *Ins1* promoter in adult mice.	[59]
	Induce reversible diabetes with β cell dysfunction but no significant dedifferentiation.	Doxycycline-inducible (Tet-Off) β cell-specific *Smad7* overexpression under the regulation of *Pdx-1* promoter in adult mice.	[58]

## Data Availability

No new data were created or analyzed in this study. Data sharing is not applicable to this article.
